# Supplementary material for: Genetics for the Women's Health Trainee: A Five-Module Curriculum
Source: MedEdPORTAL. 2019 Jan 18;15:10797. doi: 10.15766/mep_2374-8265.10797 (PMC6376891; doi:10.15766/mep_2374-8265.10797)
Supplement: Supplementary file 1 — A. Welcome Email.docx B. Objectives and Readings.docx C. Cases Only.docx D. Cases With Answers.docx E. CREOG Objectives.docx F. ACGME Milestones.docx G. End-of-Modules Feedback Form.docx [file mep-15-10797-s001.zip › _Educational Summary Report_10797.pdf]

MedEdPORTAL®
The Journal of Teaching and Learning Resources

Original Publication

OPEN ACCESS

Genetics for the Women's Health Trainee: A Five-Module Curriculum

Sarah K. Dotters-Katz, MD, MMHPE*, Neeta Vora, MD, Jeffrey Kuller, MD
*Corresponding author: sd132@duke.edu

Citation: Dotters-Katz SK, Vora N, Kuller J. Genetics for the women's health trainee: a five-module curriculum. *MedEdPORTAL*. 2019;15:10797. https://doi.org/10.15766/mep_2374-8265.10797

Copyright: © 2019 Dotters-Katz et al. This is an open-access article distributed under the terms of the Creative Commons Attribution license.

Appendices

A. Welcome Email.docx
B. Objectives and Readings .docx
C. Cases Only.docx
D. Cases With Answers.docx
E. CREOG Objectives.docx
F. ACGME Milestones.docx
G. End-of-Modules Feedback Form.docx

All appendices are peer reviewed as integral parts of the Original Publication.

Abstract

Introduction: Genetics is ubiquitous in OB-GYN. However, data suggest that trainees feel underprepared to counsel patients about genetic testing, the nuances of which are becoming increasingly complicated. We sought to develop and implement a genetics curriculum for OB-GYN residents. **Methods:** This five-module (screening for fetal aneuploidy, prenatal diagnostic testing, prenatal carrier screening, pedigrees, and cancer genetics), interactive, case-based curriculum is linked to Council on Resident Education in Obstetrics and Gynecology objectives and can stand alone or work as part of an ultrasound or obstetrics rotation. Each module, containing objectives, assigned readings, and cases with answers, is used in a small-group format and can be completed in 20-30 minutes prior to the start of a clinical day. Modules were implemented at two academic centers with first-year OB-GYN residents. Qualitative real-time feedback and summative quantitative feedback from OB-GYN residents were obtained. **Results:** Twenty-one OB-GYN residents completed the curriculum, which was well received by trainees and program directors. All residents (100%) felt the curriculum increased knowledge of prenatal genetics and felt more comfortable counseling patients after completion. Seventy-three percent enjoyed the discussion/case-based format; associated articles were found helpful by 100% of trainees. Facilitators enjoyed teaching the curriculum and felt learner knowledge improved dramatically. **Discussion:** These low-cost modules were easy to implement and resulted in increased knowledge and confidence in prenatal and cancer genetics. Designed to stand alone and take as little as 20 minutes, the modules provide a helpful adjunct to a women's health rotation or didactic curriculum.

Keywords

Genetics, Prenatal Genetics, OB-GYN, Cancer Genetics, Aneuploidy Screening, Carrier Screening

Educational Objectives

By the end of this activity, learners will be able to:

1. Counsel a patient comfortably on the basic techniques, as well as risks and benefits, of prenatal aneuploidy screening and diagnostic testing during a routine prenatal visit (measured by learner self-report).
2. Describe routine carrier screening during pregnancy and how this corresponds to clinical practice (measured by self-described knowledge acquisition on postmodule feedback survey).
3. Demonstrate an understanding of hereditary cancer syndromes as they pertain to the care of the female patient throughout her life cycle (measured by learner self-report on postmodule feedback survey).

Introduction

On a daily basis, providers caring for women during their reproductive years and beyond are incorporating genetics into practice and care for women, be it screening in pregnancy, testing for hereditary cancers, or other aspects. However, multiple authors have shown that providers of women's health constantly feel undertrained and underprepared to counsel, order, and manage results of genetic screening and testing.[1-3]

The Council on Resident Education in Obstetrics and Gynecology (CREOG) has numerous objectives related to genetic topics and includes multiple genetics-related questions on its annual in-service CREOG exam.[4] Similarly, the Accreditation Council for Graduate Medical Education (ACGME) has included milestones related to genetics.[5] Thus, the governing bodies clearly recognize the importance of learning about and gaining competency in these topics. Unfortunately, a gap still exists.

Currently, two published studies of genetic curricula for OB-GYN residents exist.[6,7] Both showed increased resident knowledge on posttests, and one also demonstrated improved resident confidence.[7] Thus, available data suggest that implementation of genetics curricula may lead to improved resident knowledge and confidence. However, teaching genetics can be challenging. Many centers do not have specialists or subject matter experts who are comfortable teaching in these areas. Additionally, evidence suggests that traditional lecture-style didactics are a less effective learning method compared to small-group discussions or case-based didactics.[8]

No formal curricula, lecture based or otherwise, exist for residencies to help teach these topics. To remedy this situation, we created a case-based, five-module, didactic curriculum relating to key topics in prenatal and cancer-based genetics and targeted at graduate medical learners who provide care for reproductive-age women. The modules can be completed either one on one or in small groups. The goal upon curriculum completion is to provide trainees the knowledge and skills to better counsel women about prenatal aneuploidy screening, prenatal diagnostic testing for aneuploidy, prenatal carrier screening, and screening/testing for hereditary cancer syndromes. These are types of counseling that any trainee providing prenatal or well-woman care should offer at every new-patient obstetric visit and at annual well-woman checks. Additionally, because dedicated genetics education can be hard to identify during OB-GYN residencies specifically, the modules are linked to the ACGME OB-GYN milestones as well as the CREOG objectives.

Within *MedEdPORTAL,* only a few publications exist that discuss prenatal screening, testing, and carrier screening.[9,10] However, none of these discuss newer screening techniques or refer specifically to management of pregnant women. More tools exist with regard to breast cancer screening, but none intended specifically for graduate medical trainees and none geared to ovarian or uterine cancer.[11-13] When we searched on the terms *lynch syndrome, ovarian cancer, endometrial cancer, endometrial carcinoma,* and *uterine cancer,* we found only one resource, which does not mention screening or genetics.[14] Thus, our case-based women's health genetics curriculum was designed to provide a resource for OB-GYN, internal medicine, and family medicine programs to teach prenatal and cancer-based genetics. This resource could also be used by other health professionals training in women's health and prenatal care, such as family nurse practitioner students, women's health nurse practitioner students, physician assistants, or midwifery students.

Methods

We developed this curriculum to increase OB-GYN interns' exposure to genetic topics at two tertiary academic centers. At both of these centers, first-year OB-GYN residents took part in an ultrasound rotation of at least 4 weeks. Because prenatal genetics is inherently tied into ultrasound, this rotation seemed the optimal time to add in a genetics curriculum. The program directors as well as the rotation directors agreed to support this implementation.

Learners did not need any specific prerequisite knowledge outside what they had learned in medical school during an OB-GYN clerkship. Although students on the OB-GYN clerkship could have been involved in these sessions, the curriculum was geared toward OB-GYN residents or possibly toward students on a fourth-year OB-GYN rotation.

Facilitators

We designed this curriculum in a case-based manner, with answers provided for the facilitator, allowing for a flipped classroom model if desired. At our two institutions, a maternal fetal medicine (MFM) geneticist and a prenatal genetic counselor led the sessions. This is not necessary, however, as the modules contain enough background/answers that a general OB-GYN, OB-GYN fellow, or chief resident could also facilitate them. Independent of who specifically led the sessions, it was ideal to have a consistent person or group of people do so for a few reasons. First, they were better able to assess a learner's progress over the block/rotation as well as to develop a rapport with the learner. Additionally, when the same people led over and over, they developed comfort with the curriculum, resulting in more consistency across the year and between learners.

Intended Audience

The target audience was OB-GYN residents. However, the modules are constructed so that they apply to any graduate medical trainees who care for pregnant women (i.e., provide prenatal care). The modules were designed to be given on a weekly basis during a rotation. For our OB-GYN residencies, this was done during the ultrasound rotation (which is in the first year).

The Modules

The curriculum comprised five modules. Each module covered a different element of prenatal or cancer genetics. Assigned prereading existed for each module along with one to three interactive cases for the learners to work through. The topics of the five modules included screening for fetal aneuploidy (Module 1), prenatal diagnostic testing (Module 2), prenatal carrier screening (Module 3), pedigrees (Module 4), and cancer genetics (Module 5). At our institution, the curriculum occurred over a 6-week rotation where there was frequently 1 week of vacation, thus one module per week. However, the other institution using this curriculum had only a 4-week rotation and did not include the cancer genetics component. Although it was helpful for Module 2 to follow Module 1, Modules 3, 4, and 5 did not require any specific order.

Introducing the Modules

We used two methods of introduce the curriculum. The first method sent an introductory email to the learner 7-10 days prior to starting the block. This email (Appendix A: genetics curriculum welcome email) introduced the learner to the curriculum and provided the objectives document (Appendix B: genetics curriculum objectives); it also contained attachments of all of the readings or a link to a central place (local drive, drop box, etc.) where the readings had been uploaded. The second method introduced the curriculum on the first day in person, explaining the expectations, readings, and where to find the information. During this session, we stressed that the learner was expected to read the articles in advance and come ready to discuss a case (or cases).

Finding a Time to Teach the Modules

We identified a prespecified teaching time for each module. For us, this was one set morning weekly for 20-30 minutes prior the start of clinic. In our setting, this was a realistic time frame for both learner and provider to arrive while still having time to cover the material prior to starting clinic. Before a busy clinic day, it might feel difficult to set aside an hour for teaching; 20 minutes was more manageable for residents and attendings alike. Also, in our setting, these were one-on-one sessions, so 60 minutes would have felt daunting for an intern. In our experience, some sessions ran longer, depending on learner curiosity, facilitator enthusiasm, and clinical factors (such as on-time arrival of the first patient). During any open-ended learning experience, the duration of the learning is dependent on many things. Each module in this curriculum was designed so that the leader could complete the cases, covering all the points, in 20 minutes. However, there are times when leaders delve deeper in certain areas, learners ask more questions, and so on, and a module may take longer in those situations. We want to emphasize, though, that the sessions are designed in such a way that if there is only 20 minutes available for teaching, the material can be effectively covered. Additionally, having a set time and location for these teaching sessions created a routine for the learner, increasing engagement.

Teaching Strategies

The curriculum was designed so that the faculty would use the associated cases (Appendices C: genetics curriculum cases and D: genetics curriculum case answers). An alternative approach that we found to be successful was to have learners review the articles, then ask questions to explore their comprehension of key concepts.

Necessary Equipment

These sessions did not require any audiovisual equipment. If such equipment is available, the cases could be displayed on a screen using PowerPoint or a word-processing program, although this is not necessary. We previously referred to emailing out materials and objective documents, which was helpful for getting them to the learner. Additionally, a shared internal drive or electronic data management system would be helpful for maintaining the resources.

Assessment

The majority of the assessment and evaluation was formative and occurred during the sessions. The faculty, through their discussions and use of cases, evaluated in real time how the learner was progressing over the course of the modules. As mentioned above, the modules were linked to CREOG objectives. In Appendix E, we list the CREOG objectives the rotation should cover and in which modules that can be accomplished. Additionally, Appendix F outlines the milestones to be assessed after completion of the modules in the setting of a clinical rotation, not as separate didactics. The facilitator reviewed these milestones and objectives prior to starting the modules.

A survey for the trainees to complete at the end of the rotation was developed to obtain feedback on the modules (Appendix G: feedback survey). The goal of this survey was not to evaluate the learner on knowledge but to assess his or her own sense of confidence, as well as to get feedback on how to improve the modules. In the past, the CREOG in-service exam had given a separate genetics score, but this score was removed in 2018. For that reason, we were unable to use this objective measure. Some published genetics quizzes exist in the literature that could also have been used for more objective assessment of knowledge.[6]

Results

These modules were first implemented at a single academic institution. The facilitators included an MFM geneticist and a prenatal genetic counselor. To date, 15 OB-GYN residents have completed the modules as part of their ultrasound rotation. Facilitators described the curriculum as “easy to implement within the confines of the rotation.” At this institution, when the rotation’s clinical locations changed, the timing and day of the week that the student met to review the articles with the facilitator were also moved. Early verbal feedback from learners at the site included the suggestion that “cases help to ‘ground’ the articles better.” Based on this feedback, the cases were created.

More recently, we adapted the curriculum and rolled it out at a second academic center. Here, the facilitator was also an MFM geneticist. In this iteration, six OB-GYN interns have completed the curriculum. They used a mix of article discussions and cases. We added the opportunity to work with a cancer genetic counselor at this site about halfway through our year.

Overall, 11 of the 21 residents completed the feedback survey. Among those, there was an even spread from both institutions. All respondents felt that the modules were helpful in increasing knowledge of prenatal genetics. Additionally, all respondents indicated that they would recommend these modules to other residents, and all felt more comfortable counseling patients about screening options for aneuploidy. Trainees felt similarly about counseling for diagnostic testing, with 73% strongly agreeing that the modules increased confidence in counseling about diagnostic testing for aneuploidy. More information about trainee responses can be found in the Table.

Table. Postcurriculum Survey Results (*n* = 11)

Statement	No. (%) Who Agreed or Strongly Agreed
The genetics curriculum was helpful in increasing my knowledge of prenatal genetics.	11 (100%)
The genetics curriculum was helpful in increasing my knowledge of cancer genetics.	3 (27%)
I would recommend this curriculum to other residents.	11 (100%)
After completing the genetics curriculum, I felt more comfortable counseling patients about screening options for aneuploidy.	11 (100%)
After completing the genetics curriculum, I felt more comfortable counseling patients about diagnostic testing for aneuploidy.	11 (100%)

Regarding Module 5 (cancer genetics), only 27% of learners found it helpful, while five out of 11 did not feel it increased knowledge about cancer genetics. Based on this feedback, we connected with our gynecologic oncology colleagues and were able to identify an oncologic genetic counselor for our trainees to work with during the rotation. Since this experience was added, learners have found it “really interesting” and “wish there was more than ½ a day.” Additional comments included “Seeing the genetic counselor in action helped to cement the cancer genetics aspects more so than the articles alone” and “[the experience] was quite valuable to see how she spoke with patients about cancer screening.”

Overall, 73% of trainees enjoyed the discussion format. Trainees felt that “the discussions were a nice way to review and learn the material” and “appreciated the time with the faculty to ask questions.” All learners who responded to the survey felt the articles were helpful, with 73% finding them very helpful. Only a third of respondents felt that the modules made the CREOG exam easier.

The feedback from the facilitators was also positive. They said that “in-person feedback from learners has been quite positive.” Facilitators also commented that “this was an easy implementation as it is built into clinical time.” Finally, one stated, “these modules add a valuable and necessary dimension to the ultrasound rotation.” Faculty also enjoyed the one-on-one time with residents. Importantly, our residency program directors appreciated that the curriculum was low cost and associated with CREOG objectives and ACGME milestones.

Discussion

For individuals training to care for women throughout their life span, genetics and genetic screening and testing play a critical role in medical management. In the setting of obstetric care, sometimes prenatal genetic counseling can feel overwhelming with all of the options for carrier and aneuploidy screening. These modules represent a low-cost, easily implementable genetics curriculum for women’s health that is linked to the CREOG objectives and ACGME milestones. The modules attempt to help the learner gain confidence regarding knowledge and counseling in these specific areas. A strength of the modules is that they can stand alone or be implemented as a set. Additionally, the modules are case based, can be used for large- or small-group didactics or as part of a broader rotation, and are linked to topically pertinent peer-reviewed articles.

Overall, our development and implementation have been relatively straightforward. Having motivated faculty facilitators has been helpful. Because our faculty were excited to teach this material, implementation was smooth. As with any curriculum, having motivated faculty makes a difference in the ease of implementation and continuation of use.

Connecting the curriculum to a rotation is helpful, as it enables the knowledge to build and be reinforced weekly. Such a connection also creates a consistent scheduled teaching time and sets expectations so that the learner is more prepared for the sessions. Time with genetic counselors (both prenatal and oncology) is valuable and is recommended as part of the modules.

Overall, for our residents who completed the modules, the curriculum helped improve their understanding of prenatal genetics. As a result, they felt more comfortable explaining the prenatal screening and diagnostic testing to patients. Genetics education had been an area of weakness for residents at both sites but now is considered a strength. Although knowledge and confidence about cancer genetics need more attention, we feel that the current exposure and education are better than what existed previously. Development and implementation of these five modules have made a difference not only in resident knowledge but also in patient care.

The biggest limitation thus far is the low number of responses on our end of-rotation-survey. In the area of trainee survey fatigue, we were plagued by a response rate slightly less than 50%. Ideally, we would have had more responses from participants about the utility of the modules. Additionally, we did not perform any summative assessment of knowledge but simply obtained trainee perceptions of knowledge and confidence. Although these were overall quite positive, we cannot say for certain that knowledge is better as a result of the modules.

As far as generalizability, we use the curriculum as part of an OB-GYN first-year resident ultrasound rotation. This could potentially limit generalizability. However, this curriculum could easily be adapted to other settings and for other trainees. For example, although some basic knowledge about prenatal genetics and its application to patient care is necessary to facilitate these sessions, a nonprenatal genetic counselor could facilitate the sessions, as could a general OB-GYN, MFM, MFM fellow, medical geneticist, OB-GYN resident, nurse midwife, or family doctor who provides women's health care. The modules could also be adapted to a residency-wide didactic curriculum, with small-group breakouts, to be led by upper level residents. In order to adapt the curriculum in this manner, we recommend the following strategy: One week prior to the session, divide the learners into groups of three to four with a spread of all trainee levels, and assign a leader, usually an upper level resident, to help run the discussion/case. We found learners had better engagement when an email with the assigned articles and cases was sent a week prior. During the didactic, the faculty leader should discuss the article in broad strokes, then have groups break out for 20 minutes of discussion. Following the team time, the facilitator should bring the groups together for review of the cases and report-back. Usually, two modules can be completed in 1 hour of didactic time. For a family medicine or non-OB-GYN residency program where trainees are learning women's health, the modules could be done during the prenatal care rotation or during an outpatient rotation with a women's health focus. Above, we have described in detail other ways which these modules could be used in the hope of expanding the audience to learners other than OB-GYN residents.

It is important to recognize that this field is rapidly changing. We have not included any sentinel papers and have tried to use society guidelines, although these too can become outdated. As genetics is a rapidly developing field, we are also limited as to the longevity of the curriculum. The final limitation of this resource is faculty to facilitate it. However, by including cases with answers, we have attempted to mitigate the need for a subject matter expert. We recognize that some level of understanding of the basic genetic principles is needed by the facilitator. However, any faculty providing prenatal care should be able to facilitate these modules. Our faculty are doing the modules one on one with residents. This is time consuming and impacts only one learner at a time. In our experience, the residents appreciate the one-on-one environment and have found it to be a strength of the curriculum. However, we have described how to use this curriculum as a didactic for either a small group (two to three learners, as one might have in clinic setting) or a large group with smaller subgroups (e.g., for a program-wide didactic).

During implementation, we identified some additional opportunities that markedly enhanced the rotation. First, we found that spending time with a genetic counselor was helpful for two reasons: Not only did it help the learner better understand the role of the genetic counselor in educating patients as well as on the health care team but also, while watching the genetic counselor interact with patients, the trainee was

provided with valuable vocabulary and phrases for his or her own counseling. Thus, while not essential, this approach can add value to the modules. Similarly, we attempted to have learners observe one amniocentesis and chorionic villus sampling. This was more doable when the modules were paired with an ultrasound rotation. Observing these procedures allowed learners to better understand what they entail and also helped them to counsel patients on the actual procedures moving forward.

We continue to use our modules at both institutions. We also continue to gather data from the end-of-rotation survey and from learners' verbal feedback verbally in order to keep improving the curriculum. We plan to work on consistency with the oncology genetic counselor, as that has been a popular adjunct to the curriculum, but scheduling can be a challenge. Some learners have asked for videos about "good counseling," and thus, we are in the process of trying to find a credible resource for our learners to watch. Finally, ideally, there would be a more objective way to assess knowledge acquisition, which we lost when the CREOG in-service exam stopped reporting the genetics scores separately. However, the concept of a test seems slightly antiquated. Thus, we are developing some self-paced vignette quizzes for learners to do each week after they meet to complete the module.

Sarah K. Dotters-Katz, MD, MMHPE: Assistant Professor, Department of Obstetrics and Gynecology, Duke University School of Medicine

Neeta Vora, MD: Associate Professor, Department of Obstetrics and Gynecology, University of North Carolina at Chapel Hill School of Medicine

Jeffrey Kuller, MD: Professor, Department of Obstetrics and Gynecology, Duke University School of Medicine

---

Disclosures
None to report.

Funding/Support
None to report.

Ethical Approval
Reported as not applicable.

References

1. Bupp CP, Demmer LA, Saul RA. Surveying the current landscape of clinical genetics residency training. *Genet Med.* 2015;17(5):386-390. https://doi.org/10.1038/gim.2014.108
2. Ready KJ, Daniels MS, Sun CC, Peterson SK, Northrup H, Lu KH. Obstetrics/gynecology residents' knowledge of hereditary breast and ovarian cancer and Lynch syndrome. *J Cancer Educ.* 2010;25(3):401-404. https://doi.org/10.1007/s13187-010-0063-4
3. Selkirk CG, Weissman SM, Anderson A, Hulick PJ. Physicians' preparedness for integration of genomic and pharmacogenetic testing into practice within a major healthcare system. *Genet Test Mol Biomarkers.* 2013;17(3):219-225. https://doi.org/10.1089/gtmb.2012.0165
4. Council on Resident Education in Obstetrics and Gynecology. *Educational Objectives: Core Curriculum in Obstetrics and Gynecology.* 10th ed. Washington, DC: American College of Obstetricians and Gynecologists; 2013.
5. The Obstetrics and Gynecology Milestone Project. *J Grad Med Educ.* 2014;6(1)(suppl 1):129-143. https://doi.org/10.4300/JGME-06-01s1-07
6. Dotters-Katz S, Hocutt G, Osborne CM, Hardisty EE, Demmer L, Vora N. Development, implementation, and assessment of a genetics curriculum across institutions. *AJP Rep.* 2016;6(4):e372-e377. https://doi.org/10.1055/s-0036-1593831
7. Macri CJ, Gaba ND, Sitzer LM, Freese L, Bathgate SL, Larsen JW Jr. Implementation and evaluation of a genetics curriculum to improve obstetrician-gynecologist residents' knowledge and skills in genetic diagnosis and counseling. *Am J Obstet Gynecol.* 2005;193(5):1794-1797. https://doi.org/10.1016/j.ajog.2005.08.003
8. Thistlethwaite JE, Davies D, Ekeocha S, et al. The effectiveness of case-based learning in health professional education. A BEME systematic review: BEME Guide No. 23. *Med Teach.* 2012;34(6):e421-e444. https://doi.org/10.3109/0142159X.2012.680939
9. Klatt E, Thompson T. Patau syndrome with holoprosencephaly: a multidisciplinary interactive case-based learning exercise. *MedEdPORTAL.* 2010;6:8181. https://doi.org/10.15766/mep_2374-8265.8181

10. Dasgupta S. Sickle cell trait and sickle cell disease: a case study. *MedEdPORTAL*. 2009;5:7920. https://doi.org/10.15766/mep_2374-8265.7920
11. Duff J, Daily K, Close J. Breast cancer instructional module. *MedEdPORTAL*. 2015;11:10023. https://doi.org/10.15766/mep_2374-8265.10023
12. Bergl PA, Feagles J. Individualizing cancer screening recommendations: a team-based learning activity for fourth-year medical students. *MedEdPORTAL*. 2017;13:10574. https://doi.org/10.15766/mep_2374-8265.10574
13. Simms-Cendan JS, Cendan JC, Boardman L, Voorhees D, Eakins MA, Merritt D. The clinical breast exam: a video and animation enhanced self-learning module. *MedEdPORTAL*. 2012;8:9131. https://doi.org/10.15766/mep_2374-8265.9131
14. Sadownik L, Finlayson S. Gyne-oncology interactive problem-based cases for postgraduate residents. *MedEdPORTAL*. 2010;6:1683. https://doi.org/10.15766/mep_2374-8265.1683

Received: September 5, 2018 | **Accepted:** December 10, 2018 | **Published:** January 18, 2019
