# Supplementary material for: Genetics for the Women's Health Trainee: A Five-Module Curriculum
Source: MedEdPORTAL. 2019 Jan 18;15:10797. doi: 10.15766/mep_2374-8265.10797 (PMC6376891; doi:10.15766/mep_2374-8265.10797)
Supplement: Supplementary file 1 — A. Welcome Email.docx B. Objectives and Readings.docx C. Cases Only.docx D. Cases With Answers.docx E. CREOG Objectives.docx F. ACGME Milestones.docx G. End-of-Modules Feedback Form.docx [file mep-15-10797-s001.zip › A. Welcome Email.docx]

Good morning,

On (INSERT YOUR DATE HERE), you will be starting your genetics curriculum. Below in this email are the learning objectives, readings, and activities, which you will be expected to complete during the curriculum.

*Please note: Do not be overwhelmed by the volume of content/information. You are not expected to complete this all at once.

Our goal for this curriculum, is for you to demonstrate an understanding of prenatal diagnosis, genetic carrier screening, and cancer genetics as they pertain to women’s health

**Expectations:**

- Each week, please read the assigned pre-reading, prior to coming to the teaching session.
- During the weekly teaching session, you will review 2-3 cases or discuss the articles with the session facilitator.

**OBJECTIVES:**

**Module 1:** Screening for aneuploidy

Objective 1: Upon completion of module 1 in the genetics curriculum, the learner will describe the basic prenatal screening techniques for aneuploidy

Objective 2: Upon completion of module 1 in the genetics curriculum, the learner will discuss the risks and benefits to the basic prenatal screening techniques for aneuploidy

Pre-Reading:

- ACOG Practice Bulletin 163 – Screening for aneuploidy
- [Grace MR](https://www.ncbi.nlm.nih.gov/pubmed/?term=Grace%20MR%5BAuthor%5D&cauthor=true&cauthor_uid=27526871) et al. Cell-Free DNA Screening: Complexities and Challenges of Clinical Implementation.

*Full citations for Readings:*

* American College of, O., Gynecologists' Committee on Practice, B.-O., Committee on, G., & Society for Maternal-Fetal, M. (2016). Practice Bulletin No. 163: Screening for Fetal Aneuploidy. (2016). *Obstet Gynecol, 127*(5), e123-137. doi:10.1097/AOG.0000000000001406

* Grace, M. R., Hardisty, E., Dotters-Katz, S. K., Vora, N. L., & Kuller, J. A. (2016). Cell-Free DNA Screening: Complexities and Challenges of Clinical Implementation. *Obstet Gynecol Surv, 71*(8), 477-487. doi:10.1097/OGX.0000000000000342

**Module 2: Prenatal diagnostic testing**

Objective 3: Upon completion of module 2 in the genetics curriculum, the learner will explain basic details, risks, and benefits, as well as time frame for amniocentesis and chorionic villus sampling

Pre-Reading:

- ACOG Committee Opinion 693: Counseling About Genetic Testing and Communication of Genetic Test Results
- ACOG Practice Bulletin 162: Prenatal Diagnostic testing for Genetic Disorders

*Full citations for Readings:*

* Committee on, G. (2017c). Committee Opinion No. 693: Counseling About Genetic Testing and Communication of Genetic Test Results. *Obstet Gynecol, 129*(4), e96-e101. doi:10.1097/AOG.0000000000002020

* American College of, O., Gynecologists' Committee on Practice, B.-O., Committee on, G., & Society for Maternal-Fetal, M. (2016). Practice Bulletin No. 162: Prenatal Diagnostic Testing for Genetic Disorders. *Obstet Gynecol, 127*(5), e108-122.

**Module 3: Prenatal Carrier Screening**

Objective 4: Upon completion of module 3 in the genetics curriculum, the learner will understand prenatal carrier screening

Pre-Reading:

- ACOG Committee Opinion 690: Carrier Screening in the age of Genomic medicine
- ACOG Committee Opinion 691: Carrier Screening for Genetic Conditions

*Full citations for Readings:*

* Committee on, G. (2017a). Committee Opinion No. 690: Carrier Screening in the Age of Genomic Medicine. *Obstet Gynecol, 129*(3), e35-e40.

* Committee on, G. (2017b). Committee Opinion No. 691: Carrier Screening for Genetic Conditions. *Obstet Gynecol, 129*(3), e41-e55. doi:10.1097/AOG.0000000000001952

**Module 4: Pedigrees**

Objective 5: Upon completion of module 4 in the genetics curriculum, the learner will describe basic patterns of genetic inheritance based on pedigrees

Pre-Reading:

- ACOG Practice Bulletin 162: Prenatal Diagnostic Testing for Genetic Disorders

*Full citations for Readings:*

* American College of, O., Gynecologists' Committee on Practice, B.-O., Committee on, G., & Society for Maternal-Fetal, M. (2016). Practice Bulletin No. 162: Prenatal Diagnostic Testing for Genetic Disorders. *Obstet Gynecol, 127*(5), e108-122. doi:10.1097/AOG.0000000000001405

**Module 5: Cancer Genetics**

Objective 6: Upon completion of module 5 in the genetics curriculum, the learner will demonstrate an understanding of hereditary cancer syndromes as they pertain to ob/gyn.

Pre-Reading:

- ACOG Practice bulletin 182: Hereditary Breast and Ovarian Cancer Syndrome
- ACOG Practice Bulletin No. 147: Lynch syndrome
- ACOG Committee opinion 634: Hereditary cancer syndromes and risk assessment

*Full citations for Readings:*

* Committee on Practice Bulletins-Gynecology, C. o. G. S. o. G. O. (2017). Practice Bulletin No 182: Hereditary Breast and Ovarian Cancer Syndrome. *Obstet Gynecol, 130*(3), e110-e126. doi:10.1097/AOG.0000000000002296

* Committee on Practice, B.-G., & Society of Gynecologic, O. (2014). ACOG Practice Bulletin No. 147: Lynch syndrome. *Obstet Gynecol, 124*(5), 1042-1054. doi:10.1097/01.AOG.0000456325.50739.72

* Committee opinion no. 634: Hereditary cancer syndromes and risk assessment. (2015). *Obstet Gynecol, 125*(6), 1538-1543. doi:10.1097/01.AOG.0000466373

On (INSERT MEETING TIME AND DAY HERE), (INSERT FACILIATATOR NAME HERE) will review the topic of the week with you. Thus, why the above documents are important.

Please let (INSERT CURRICULUM DIRECTOR HERE) know if you have any questions / concerns regarding the genetics.

Thank you. We look forward to working with you.

(INSERT CURRICULUM LEADER NAME AND EMAIL HERE)

Attachments:

(INSERT A LINK TO YOUR DROPBOX OR LOCAL DRIVE HERE)
