# Supplementary material for: Genetics for the Women's Health Trainee: A Five-Module Curriculum
Source: MedEdPORTAL. 2019 Jan 18;15:10797. doi: 10.15766/mep_2374-8265.10797 (PMC6376891; doi:10.15766/mep_2374-8265.10797)
Supplement: Supplementary file 1 — A. Welcome Email.docx B. Objectives and Readings.docx C. Cases Only.docx D. Cases With Answers.docx E. CREOG Objectives.docx F. ACGME Milestones.docx G. End-of-Modules Feedback Form.docx [file mep-15-10797-s001.zip › B. Objectives and Readings.docx]

**Genetics Curriculum**

**Overall Goal:** Upon completion of this genetics curriculum, the learner will demonstrate an understanding of prenatal diagnosis, genetic carrier screening, and cancer genetics as they pertain to women’s health

**Expectations:**

- Each week, please read the assigned pre-reading, prior to coming to the teaching session.
- During the weekly teaching session, you will review 2-3 cases or discuss the articles with the session facilitator.

**Module 1:** Prenatal Screening for aneuploidy

Objective 1: Upon completion of module 1 in the genetics curriculum, the learner will apply the basic prenatal screening techniques for aneuploidy in counseling patients in clinic and/or during the discussion with the facilitator

Objective 2: Upon completion of module 1 in the genetics curriculum, the learner will discuss the risks and benefits to the basic prenatal screening techniques for aneuploidy with the facilitator

Pre-Reading:

- ACOG Practice Bulletin 163 – Screening for aneuploidy
- [Grace MR](https://www.ncbi.nlm.nih.gov/pubmed/?term=Grace%20MR%5BAuthor%5D&cauthor=true&cauthor_uid=27526871) et al. Cell-Free DNA Screening: Complexities and Challenges of Clinical Implementation.

Full citations for Readings:

* American College of, O., Gynecologists' Committee on Practice, B.-O., Committee on, G., & Society for Maternal-Fetal, M. (2016). Practice Bulletin No. 163: Screening for Fetal Aneuploidy. (2016). *Obstet Gynecol, 127*(5), e123-137. doi:10.1097/AOG.0000000000001406

* Grace, M. R., Hardisty, E., Dotters-Katz, S. K., Vora, N. L., & Kuller, J. A. (2016). Cell-Free DNA Screening: Complexities and Challenges of Clinical Implementation. *Obstet Gynecol Surv, 71*(8), 477-487. doi:10.1097/OGX.0000000000000342

**Module 2: Prenatal diagnostic testing**

Objective 3: Upon completion of module 2 in the genetics curriculum, the learner will counsel (a theoretical patient) on the basic details, risks, and benefits, as well as time frame for amniocentesis and chorionic villus sampling with the facilitator observing

Pre-Reading:

- ACOG Committee Opinion 693: Counseling About Genetic Testing and Communication of Genetic Test Results
- ACOG Practice Bulletin 162: Prenatal Diagnostic testing for Genetic Disorders

Full citations for Readings:

* Committee on, G. (2017c). Committee Opinion No. 693: Counseling About Genetic Testing and Communication of Genetic Test Results. *Obstet Gynecol, 129*(4), e96-e101. doi:10.1097/AOG.0000000000002020

* American College of, O., Gynecologists' Committee on Practice, B.-O., Committee on, G., & Society for Maternal-Fetal, M. (2016). Practice Bulletin No. 162: Prenatal Diagnostic Testing for Genetic Disorders. *Obstet Gynecol, 127*(5), e108-122.

**Module 3: Prenatal Carrier Screening**

Objective 4: Upon completion of module 3 in the genetics curriculum, the learner will utilize prenatal carrier screening in prenatal care

Pre-Reading:

- ACOG Committee Opinion 690: Carrier Screening in the age of Genomic medicine
- ACOG Committee Opinion 691: Carrier Screening for Genetic Conditions

Full citations for Readings:

* Committee on, G. (2017a). Committee Opinion No. 690: Carrier Screening in the Age of Genomic Medicine. *Obstet Gynecol, 129*(3), e35-e40.

* Committee on, G. (2017b). Committee Opinion No. 691: Carrier Screening for Genetic Conditions. *Obstet Gynecol, 129*(3), e41-e55. doi:10.1097/AOG.0000000000001952

**Module 4: Pedigrees**

Objective 5: Upon completion of module 4 in the genetics curriculum, the learner will deduce basic patterns of genetic inheritance based on pedigrees using the associated modules

Pre-Reading:

- ACOG Practice Bulletin 162: Prenatal Diagnostic Testing for Genetic Disorders

Full citations for Readings:

* American College of, O., Gynecologists' Committee on Practice, B.-O., Committee on, G., & Society for Maternal-Fetal, M. (2016). Practice Bulletin No. 162: Prenatal Diagnostic Testing for Genetic Disorders. *Obstet Gynecol, 127*(5), e108-122. doi:10.1097/AOG.0000000000001405

**Module 5: Cancer Genetics**

Objective 6: Upon completion of module 5 in the genetics curriculum, the learner will describe various of hereditary cancer syndromes as they pertain to ob/gyn during discussion with a facilitator or genetic counselor.

Pre-Reading:

- ACOG Practice bulletin 182: Hereditary Breast and Ovarian Cancer Syndrome
- ACOG Practice Bulletin No. 147: Lynch syndrome
- ACOG Committee opinion 634: Hereditary cancer syndromes and risk assessment

Full citations for Readings:

* Committee on Practice Bulletins-Gynecology, C. o. G. S. o. G. O. (2017). Practice Bulletin No 182: Hereditary Breast and Ovarian Cancer Syndrome. *Obstet Gynecol, 130*(3), e110-e126. doi:10.1097/AOG.0000000000002296

* Committee on Practice, B.-G., & Society of Gynecologic, O. (2014). ACOG Practice Bulletin No. 147: Lynch syndrome. *Obstet Gynecol, 124*(5), 1042-1054. doi:10.1097/01.AOG.0000456325.50739.72

* Committee opinion no. 634: Hereditary cancer syndromes and risk assessment. (2015). *Obstet Gynecol, 125*(6), 1538-1543. doi:10.1097/01.AOG.0000466373

Optional Additional activities (if possible based on location):

1. Please complete the tutorial: <http://brighter-tomorrows.org/>
2. Observe at least one amniocentesis and chorionic villus sampling
3. Spend 2-3 sessions with a prenatal genetic counselor – including at least one for Advanced maternal age, at least one for first trimester screen, and at least one for carrier screen discussion
4. Spend ½ day with a cancer genetic counselor
