# Supplementary material for: Genetics for the Women's Health Trainee: A Five-Module Curriculum
Source: MedEdPORTAL. 2019 Jan 18;15:10797. doi: 10.15766/mep_2374-8265.10797 (PMC6376891; doi:10.15766/mep_2374-8265.10797)
Supplement: Supplementary file 1 — A. Welcome Email.docx B. Objectives and Readings.docx C. Cases Only.docx D. Cases With Answers.docx E. CREOG Objectives.docx F. ACGME Milestones.docx G. End-of-Modules Feedback Form.docx [file mep-15-10797-s001.zip › C. Cases Only.docx]

Genetics Cases

**Week one: Prenatal screening**

Case 1: A 27yo wishes to discuss options for prenatal screening

1. What are her options?
   1. How does her gestational age effect those options?
2. Would you offer her diagnostic testing? Would you recommend this?
3. If she is in the 1^st^ trimester, which test would you recommend?
   1. What if her ultrasound shows twins? Does this change her options?
   2. What if she has a prior child with trisomy 21, does this change her options?
   3. If she elects for 1^st^ trimester screening, is there any other screening that you would recommend in the 2^nd^ trimester?
4. What if she is 18 weeks, which test would you recommend?
   1. What does this screen for?

Case 2: A 41 yo wishes to discuss options for prenatal screening

1. What are her options?
   1. How does her gestational age effect those options?
2. Would you offer her diagnostic testing? Would you recommend this?
3. If she was 7 weeks, which test would you recommend?
   1. What if her ultrasound shows twins? Does this change her options?
   2. What if she has a prior child with trisomy 21, does this change her options?
4. She elects for cfDNA.
   1. At what gestational age can you use cfDNA?
   2. If it returns as low fetal fraction, what are possible explanations for this? How would you counsel her?
   3. If it returns with multiple aneuploidies, what are possible explanations for this? How would you counsel her?
   4. If it returns with Trisomy 21, what are possible explanations for this? How would you counsel her?

**Week 2: Prenatal Diagnosis**

Case 1: A 37yo patient is referred to you with a cfDNA positive for T21.

1. What does this result mean? Talk me through what you would say to her in counseling.
2. What are her diagnostic options?
   1. Explain what a CVS entails. What are the risks? At what gestational age can she have this done?
   2. Explain what an amniocentesis entails. What are the risks? At what gestational age can she have this done?
3. Does the gestational age at which she receives the cfDNA results impact her options?
   1. How would you counsel her if she is 22 weeks?
   2. What if she is 11 weeks?

Case 2: A low risk 22yo comes in for a first trimester screen.

1. At that time, there is a large cystic hygroma seen.
   1. What is your differential diagnosis? What is the likelihood of each of these?
   2. What are her options?
   3. How would you counsel her?
2. She elects for a CVS.
   1. If it returns normal, what is the most likely etiology now?
      1. What other testing does she need? How would you counsel her?
   2. If it returns with Trisomy 21, what is the most likely etiology now?
      1. What other testing does she need? How would you counsel her?

**Week 3: Carrier screening**

Case 1: A 24yo G1 comes in for a new OB visit. She is of African descent.

1. What carrier screening would you offer her?
   1. How would you counsel her about the risks of this test?
   2. Does her race / ethnicity effect this?
2. Her electrophoresis returns with some HgbS present, how would you interpret this result?
   1. What are the next steps? What test does her partner need to have done?
   2. If his test returns the same as hers, what is the risk that the fetus is has sickle cell disease?
   3. If her partner cannot be tested, but he was African American male, what is the chance that he was a carrier for sickle cell disease?

Case 2: A 18yo G1 comes for a new OB visit. She has recently moved from Quebec, where her family lived for 4 generations.

1. What carrier screening would you offer her?
   1. How would you counsel her about the risks of this test?
2. She comes back as a carrier for cystic fibrosis (CF).
   1. What is the general carrier frequency for this condition? What about for individuals of her ethnicity?
   2. Does this result impact her?
3. What are the next steps?
   1. If her partner is a carrier, what is the risk that her fetus will have CF? A carrier?
   2. What if her partner is a carrier of a different mutation? How does this effect fetal risk?

Case 3: A 36yo G1 comes in for her new OB visit. She is Jewish, as is her partner.

1. What screening would you offer her based on her ethnicity?
   1. How would you counsel her about the risks of this test?
2. What genetic conditions are on that panel?
   1. List the carrier frequency of those.
   2. Describe the basic mutation / genetic problem of each of those syndromes.

**Week 4: Pedigree analysis / patterns of inheritance**

Case 1: Please use the pedigree below to complete the case


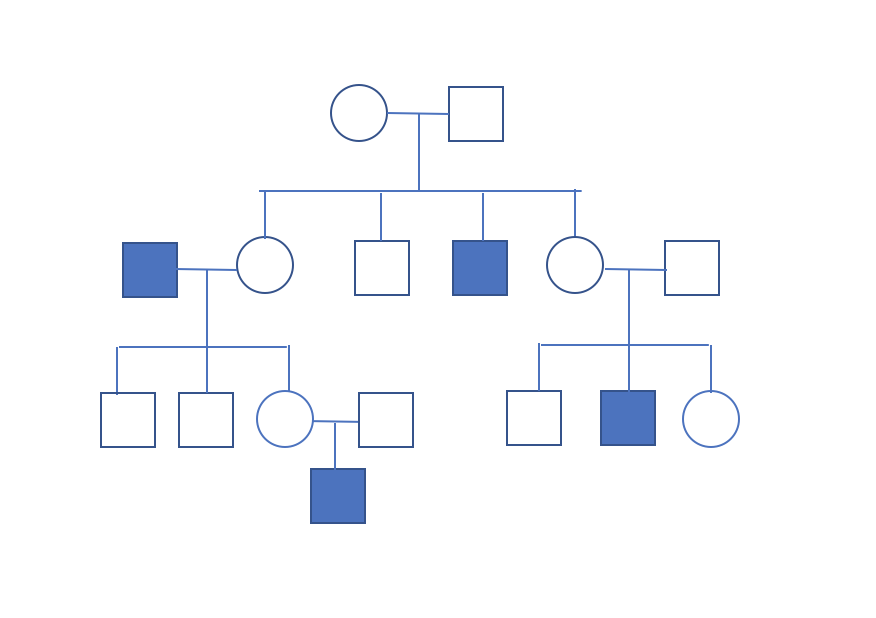


1. A 25yo G1 comes to see you in preconception counseling. This is her pedigree.
   1. What type on condition are you concerned about?
      1. What in the pedigree makes you think this?
      2. What are some examples of genetic disorders that are inherited this way?
   2. How you counsel her about her risk of having the disorder? Could she be a carrier?
   3. What about a fetus? Does the gender of the fetus impact the risk?
   4. Are there options for genetic diagnosis of a pregnancy? What are they?

Case 2: Please use the pedigree below to complete the case.


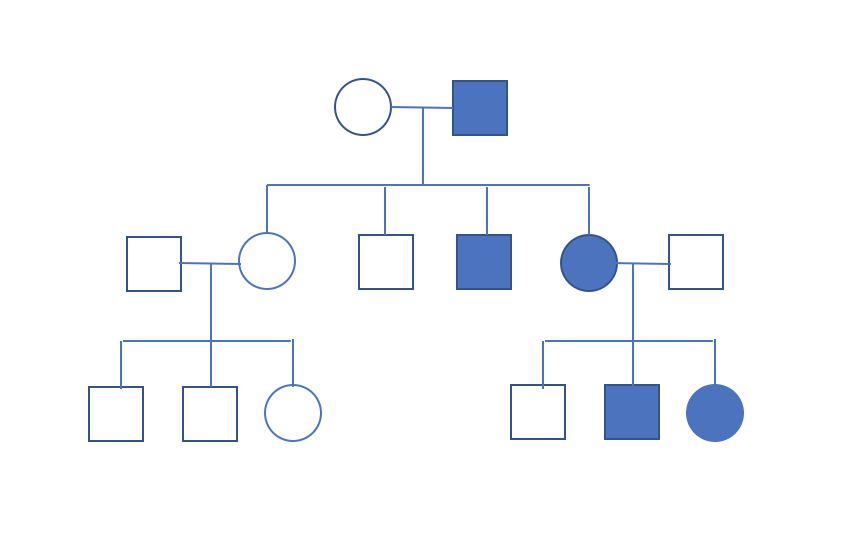


1. A 27yo G1 comes to see you in preconception counseling. This is her partner’s pedigree (single arrow).
   1. What type on condition are you concerned about?
      1. What in the pedigree makes you think this?
      2. What are some examples of genetic disorders that are inherited this way?
   2. How you counsel her about partner’s risk of having the disorder? Could he be a carrier? What about a fetus?
   3. Are there options for genetic diagnosis during pregnancy? What are they?
2. What if her partner is the double arrow?
   1. How you counsel her about her fetus’ risk of having the disorder? Does the gender of the fetus matter?

**Week 5: Cancer Genetics**

Case 1: 35 yo female presents as a new patient for her GYN annual exam.

1. You ask about her family history, which is significant for breast cancer in her mom and maternal aunts. Her maternal great aunts both also died of breast cancer.
   1. What are the red flags in her history?
   2. What other data about these cancers might be helpful?
   3. What genetic mutations are you worried about?
      1. How would you screen / test for these?
2. After seeing you, the above patient talks to her mom more about the cancer. It turns out that her mom was tested and her mom has a BRCA2 mutation. The patient elects to be tested herself. Her test returns positive for the BRCA2 mutation.
   1. When does the patient need to start mammograms? What kind does the patient need?
   2. Does the patient need other surveillance?
   3. How would you counsel the patient about her risks of breast cancer? Other cancers?
   4. How would you counsel the patient regarding management of her ovaries / tubes?
3. If her mom was tested for BRCA and was negative for a BRCA mutation, what breast cancer screening regimen would you recommend for your patient?

Case 2: 53yo comes in for her annual exam.

1. In the last year, she had a maternal aunt diagnosed with endometrial cancer and her mother died of colon cancer.
   1. What other family history might be helpful in figuring out if there is a hereditary cancel syndrome present?
      1. What cancer syndromes are on your differential diagnosis?
      2. Describe each of these, what cancers are seen, and what are the molecular mechanisms of these syndromes?
      3. How would you diagnose each of these?
      4. What screening would you recommend for her if she does not appear to have a hereditary cancer syndrome? If she does?
