# Supplementary material for: Genetics for the Women's Health Trainee: A Five-Module Curriculum
Source: MedEdPORTAL. 2019 Jan 18;15:10797. doi: 10.15766/mep_2374-8265.10797 (PMC6376891; doi:10.15766/mep_2374-8265.10797)
Supplement: Supplementary file 1 — A. Welcome Email.docx B. Objectives and Readings.docx C. Cases Only.docx D. Cases With Answers.docx E. CREOG Objectives.docx F. ACGME Milestones.docx G. End-of-Modules Feedback Form.docx [file mep-15-10797-s001.zip › D. Cases With Answers.docx]

Genetics Cases

Week one: Prenatal screening

Case 1: A 27yo wishes to discuss options for prenatal screening for aneuploidy

1. **What are her options?**

| Option | Components | Gestational age | Screens for… |
| --- | --- | --- | --- |
| LOW RISK OPTIONS | | | |
| First trimester screen | Maternal serum beta human chorionic gonadotropin (beta-hCG), pregnancy-associated plasma protein-A (PAPP-A) and Ultrasound measurement of nuchal translucency (NT)* | 11 0/7 to 13 6/7 | Trisomy 13, 18, 21 |
| Integrated screen | Maternal serum PAPP-A and an ultrasound measurement of NT* AND later adds maternal serum alpha fetoprotein [AFP], unconjugated estriol [uE3], inhibin A and beta-hCG^a^ | 10 0/7 to 13 6/7, then second set drawn in 2^nd^ trimester – all markers put together to calculate risk | Trisomy 13,18, 21 and open neural tube defects (ONTD) |
| Sequential Screen | Maternal serum PAPP-A and an ultrasound measurement of NT* – if not elevated, then adds maternal serum alpha fetoprotein [AFP], unconjugated estriol [uE3], inhibin A and beta-hCG^a^ and reassesses risk | 10 0/7 to 13 6/7 – if not elevated risk, then second set drawn in 2^nd^ trimester – markers combined to recalculate risk | Trisomy 13,18, 21 and ONTD |
| Quad or Tetra Screen | Maternal serum AFP, uE3, inhibin A and beta-hCG^a^ | 15 0/7 weeks to 18 6/7 weeks optimally, but can be done as late as 22 6/7 | Trisomy 13,18, 21 and ONTD |
| HIGH RISK OPTIONS | | | |
| Cell free DNA (aka noninvasive prenatal testing, NIPT or noninvasive prenatal screening NIPS) | Circulating cell free DNA from maternal and fetal-placental units (serum test) | 10 0/7 until delivery | Trisomy 13,18,21, and sex chromosome aneuploidies |

*NT may also screen for Monosomy X and triploidy

^a^ The 2^nd^ trimester serum markers can also screen for Smith-Lemli-Opitz

- 1. **How does her gestational age effect those options?**

See table above

1. **Would you offer her diagnostic testing?**

We offer diagnostic testing to all women, regardless of risk. Her baseline age related risk is quite low. However, if her anxiety level was really high, could offer this to her.

1. **If she is in the 1^st^ trimester, which test would you recommend?**

1^st^ trimester screen has the highest detection rate of tests in the 1^st^ trimester alone. Integrated and sequential screens have lower false positive rates, but must be contain 2^nd^ trimester components to lower that rate.

- 1. **What if her ultrasound shows twins? Does this change her options?**

In the setting of twins, during a first trimester screen, each fetus gets its own NT measurement. Thus, a separate risk profile can be calculated for each twin. For serum-based tests, it is harder to delineate between the twins. Overall, serum levels may be higher. However, this assumes an equal contribution from each fetus, which may not be the case. Consider if one twin had a contributed a higher than average amount of inhibin while the other twin contributed lower than average. The sum might appear to represent an average amount for each fetus; thus, blunting the risk profile and possibly causing a false negative. In the In the case of monozygotic twins, serum-based tests are likely to perform better than dizygotic cases because if there is a chromosomal problem it effects both fetuses.

- 1. **What if she has a prior child with trisomy 21, does this change her options?**

With a prior affected child, she becomes high risk. Her options change; thus diagnostic testing is a much more reasonable choice as well. (See “high risk options” in table above)

- 1. **If she elects for 1^st^ trimester screening, is there any other screening that you would recommend in the 2^nd^ trimester?**

The first trimester screen does not assess ONTD risk. In this case, we offer AFP-only. In reality, many patients decline AFP-only as within a few weeks they receive an anatomic survey, which also effectively screens for ONTD.

1. **What if she is 18 weeks, which test would you recommend?**

At this gestational age, she is eligible for a quad screen. The other three low-risk screening tests all require some means of first trimester assessment.

- 1. **What does this screen for?**

See table above

Case 2: A 41 yo wishes to discuss options for prenatal screening

1. **What are her options?**

Women who are high risk can elect for any of the low-risk screening options, but the high-risk screening options are recommended (see table above). They can, of course, also have diagnostic testing. High-risk women include those age 35 and over, women with a prior trisomic pregnancy, a fetus with at least one major or two minor anomalies, and a couple where one partner has a chromosomal translocation, inversion, or aneuploidy.

- 1. **How does her gestational age effect those options?**

cfDNA can be done at any gestational age that low-risk screening can be done.

1. **Would you offer her diagnostic testing?**

All women should be offered diagnostic testing.

1. **If she was 7 weeks, which test would you recommend?**

No screening tests can be done this early. She will have to wait until after 10 weeks for a screening test.

- 1. **What if her ultrasound shows twins? Does this change her options?**

There are some cfDNA companies which offer cfDNA for twins. However, at this time ACOG and ACMG do not endorse or recommend this option. Thus, a 1^st^ screen is the best test in the setting of twins.

- 1. **What if she has a prior child with trisomy 21, does this change her options?**

A prior child with trisomy 21 is included as a high-risk criterion. Thus, we recommend cfDNA.

1. **She elects for cfDNA.**
   1. **At what gestational age can you use cfDNA?**

See table above

- 1. **If it returns as low fetal fraction, what are possible explanations for this? How would you counsel her?**

Low fetal fraction can be due to early gestational age at collection, suboptimal sample collection or handling, high maternal BMI, use of low molecular weight heparin, and fetal karyotype abnormalities. She needs to be counseled on the various possibilities for this. She should also be offered ultrasound to confirm gestational age as well as redraw of the lab. She would also be offered diagnostic testing.

*Of note, women with high BMI have a much higher rate of low fetal fraction. Thus, this result is more likely to represent a euploid fetus in a women with a BMI of 55 than a BMI of 25. In other words, a woman with a BMI of 25 and a cfDNA that returns with low fetal fraction is more likely to have a fetus with T21, than a woman who’s BMI is 55 with the same lab result.

- 1. **If it returns with multiple aneuploidies, what are possible explanations for this? How would you counsel her?**

In this setting, there is concern for maternal cancers, confined placental mosaicism, and fetal aneuploidy. The patient needs to be counseled on these outcomes and evaluation for maternal cancer should be considered. She should also be offered diagnostic testing.

- 1. **If it returns with Trisomy 21, what are possible explanations for this? How would you counsel her?**

Cell free DNA is incredibly sensitive in high risk populations, with detection rates as high as 99.5% and false positive rates of 0.05%. In her case, as she is high-risk, this most likely represents a true positive. However, the cell free DNA is not a diagnostic test. Confirmatory diagnostic testing should be strongly RECOMMENED. Though a vanishing twin and confined placental mosaicism can lead to this result, in a patient who is high risk a priori, these are less likely. Of note, confined placental mosaicism is seem more commonly with T13 than with T21.

Week 2: Prenatal Diagnosis

Case 1: A 37yo patient is referred to you with a cfDNA positive for T21.

1. **What does this result mean?**

Because she is high-risk to start with, this likely represents a true positive. However, this is only a screening test. Thus, we would offer and recommend a diagnostic test.

1. **What are her diagnostic options?**

There are two diagnostic testing options – chorionic villus sampling (CVS) and amniocentesis (also referred to as amnio).

- 1. **Explain what a CVS entails. What are the risks? At what gestational age can she have this done?**

CVS stands for chorionic villus sampling. This test can be thought of as a placental biopsy to obtain cells for genetic testing (usually aneuploidy, though can be for many genetic conditions). The procedure may be done trans-cervically or transabdominally depending on surgeon and placental location. It is done in the office and the patient does not require any anesthesia. The procedure itself is done under ultrasound guidance and takes less than about 5-10 minutes. CVS can be done from 10 0/7 weeks to 13 6/7 weeks. The risks include bleeding, infection, membrane rupture, labor, and pregnancy loss (~1/500).

- 1. **Explain what an amniocentesis entails. What are the risks? At what gestational age can she have this done?**

Amniocentesis (amnio) entails inserting a needle through the uterus into the amniotic fluid to remove 10-30cc of fluid. In the fluid, are fetal cells which can be used for genetic testing. This is done transabdominally. It is often done in the office, though after 24 weeks, should be done on labor and delivery. Risks include bleeding, infection, rupture of membranes, labor and pregnancy loss (~1/1000). This is usually done after 16 0/7 weeks. The procedure takes only a few minutes.

1. **Does the gestational age at which she receives the cfDNA results impact her options?**

Her gestational age impact what diagnostic testing she can have. It may also impact her ability to terminate the pregnancy if she desires that option.

- 1. **How would you counsel her if she is 22 weeks?**

At 22 weeks, she can have an amniocentesis.

- 1. **What if she is 11 weeks?**

At 11 weeks, she is a candidate for CVS. She can also wait until 16+ weeks to have an amniocentesis.

Case 2: A low risk 22yo comes in for a first trimester screen.

1. **At that time, there is a large septated cystic hygroma seen.**
   1. **What is your differential diagnosis? What is the likelihood of each of these?**

| Diagnosis | Likelihood |
| --- | --- |
| Aneuploidy (Trisomy 21, Monosomy X, and others) | ~50% |
| Major anomaly (skeletal or cardiac) | ~30% of remaining (those not chromosomal) OR ~16% of total |

Of the remaining normal fetuses, about 8% will have an unexplained IUFD. This represents a 6-fold increase risk from baseline. Overall, 17% of fetuses with septate cystic hygroma have normal pediatric outcomes.

- 1. **What are her screening and diagnostic testing options?**

The recommended test is a diagnostic test. At this gestational age, she can have a CVS. She could also wait and have amniocentesis. Some patients do not want the risk of fetal loss associated with diagnostic testing. In these cases, cfDNA can be offered. However, it is CRITICAL to emphasize that this is not a diagnostic test and that tests for major chromosomal problems. In other words, cfDNA does not assess look at all chromosomes in the way that a karyotype from a diagnostic test can.

- 1. **How would you counsel her?**

We recommend diagnostic testing with either amnio or CVS. If she declines these, then we could offer cfDNA.

*NB: Actual discussion would vary. (We recommend having learners talk through what they would say to a patient as if you were the patient.)

1. **She elects for a CVS.**
   1. **If it returns normal, what is the most likely etiology now?**

The most likely etiology now is a major cardiac or skeletal anomaly. A genetic syndrome is also possible.

- - 1. **What other fetal assessment does she need? How would you counsel her?**

We would recommend a detailed anatomic survey as well as a fetal echo.

*NB: Actual discussion would vary. (We recommend having learners talk through what they would say to a patient as if you were the patient.)

- 1. **If it returns with Trisomy 21, what is the most likely etiology now?**

At this point – she now has diagnostic testing consistent with Trisomy 21, giving this fetus that diagnosis.

- - 1. **What other fetal assessment does she need? How would you counsel her?**

We would recommend a detailed anatomic survey as well as a fetal echo. Serial assessment of fetal growth and antenatal testing would depend on other anomalies identified and vary by center.

*NB: Actual discussion would vary. (We recommend having learners talk through what they would say to a patient as if you were the patient.)

Week 3: Carrier screening

**Case 1: A 24yo G1 comes in for a new OB visit. She is of African descent.**

1. **What carrier screening would you offer her?**

ACOG recommends that all patients be offered screening for cystic fibrosis and spinal muscular atrophy– independent of race and ethnicity. Additionally, ACOG recommends a complete blood count for all women, and if abnormal, a hemoglobin electrophoresis. For women of African, Mediterranean, Middle Eastern, Southeast Asian, or West Indian descent then hemoglobin electrophoresis should be performed with the complete blood count.

- 1. **How would you counsel her about the risks of this test?**

These tests are low risk to her and the pregnancy. When positive, the tests may provide information which could lead to further testing or diagnosis for herself, her partner, and her fetus. However, it is important to note that they do not detect all mutations.

- 1. **Does her race / ethnicity effect this?**

Because she is of African descent we would recommend hemoglobin electrophoresis.

1. **Her electrophoresis returns with some HgbS present but more Hbg A present, how would you interpret this result?**

This results is consistent with sickle cell trait. In other words, she is carrier for the sickle beta globin mutation.

- 1. **What are the next steps? What test does her partner need to have done?**

Women with sickle cell trait are at higher risk for urinary tract infection during pregnancy. Thus, from a pregnancy perspective, the patient should have urine cultures each trimester. There is also a 50% chance she will pass this gene on to her fetus. Her partner can be tested similarly.

- 1. **If his test returns the same as hers, what is the risk that the fetus is affected? What is the risk that the fetus would be a carrier?**

If both parents are carriers, there is a 25% chance that the fetus will be affected and a 50% chance he/she will be a carrier.

- 1. **If her partner cannot be tested, but he was African American male, what is the chance that he was a carrier for sickle cell disease?**

Prevalence of Sickle cell trait in African Americans is about 7-10%.

Case 2: A 18yo G1 comes for a new OB visit. She has recently moved from Quebec, where here family had lived for 4 generations.

1. **What carrier screening would you offer her?**

ACOG recommends that all patients be offered screening for cystic fibrosis and spinal muscular atrophy– independent of race and ethnicity. Additionally, ACOG recommends a complete blood count for all women, and if abnormal, a hemoglobin electrophoresis. In her case, we also would also offer screening for Tay-Sachs disease.

- 1. **How would you counsel her about the risks of this test?**

These tests are low risk to her and the pregnancy. When positive, the tests may provide information which could lead to further testing or diagnosis for herself, her partner, and her fetus. However, it is important to note that they do not detect all mutations.

1. **She comes back as a carrier for Cystic Fibrosis (CF).**
   1. **What is the general carrier frequency for this condition?**

The general carrier frequency for CF is 1/24.

- 1. **What about for individuals of her ethnicity?**
  2. Among French Canadians, the carrier frequency is between 1/15-1/20. Some Canadian populations have a carrier frequency as high as 1/5. There are specific CF panels for Canadians.
  3. **Does this result affect her?**

Most CF carrier are asymptomatic. In the majority of cases, being a CF carrier does not have medical implications. However, there may be implications to her fetus.

1. **What are the next steps?**

The next step is to offer expanded screening to her partner, called a Pan-ethnic panel. Of note, for high risk couples or women, it is reasonable to test both partners at the same time to expedite results.

- 1. **If her partner is a carrier, what is the risk that her fetus will be affected? A carrier?**

Cystic fibrosis is inherited in an autosomal recessive fashion. If both parents are carriers, there is a 25% chance that the fetus will be affected and a 50% chance he/she will be a carrier.

- 1. **What if her partner is a carrier of a different mutation? How does this effect fetal risk?**

The exact phenotype of an affected fetus/child will depend on what mutations each parent has. The numerical risks are the same, but the severity of the disease may differ.

Of note, as there are over 1700 different mutations in CF, there are cases where a patient has a family history of disease but her screening tests returns negative.

Case 3: A 36yo G1 comes in for her new OB visit. She is Jewish as is her partner.

1. **What screening would you offer her based on her ethnicity?**

ACOG recommends that all patients be offered screening for cystic fibrosis and spinal muscular atrophy– independent of race and ethnicity. Additionally, ACOG recommends a complete blood count for all women, and if abnormal, a hemoglobin electrophoresis. In her case, we would also offer Canavan disease, Cystic fibrosis, Familial dysautonomia and Tay–Sachs disease. There is a more extensive “Ashkenazi” panel, that some providers might offer as well.

- 1. **How would you counsel her about the risks of this test?**

These tests are low risk to her and the pregnancy. When positive, the tests may provide information which could lead to further testing or diagnosis for herself, her partner, and her fetus. However, it is important to note that they do not detect all mutations.

1. **What conditions are on that panel?**

| Condition | Carrier Frequency | Mutation | “Problem” |
| --- | --- | --- | --- |
| Cystic Fibrosis | 1/19-29 | Mutation in CFTR gene | Respiratory and GI issues |
| Bloom Syndrome | 1/102 | Mutation in BLM gene | Short stature, increased cancer risk |
| Canavan Disease | 1/40 | Deficiency of the enzyme aspartoacylase (ASPA) | Early onset neurodegenerative disease |
| Familial dysautonomia | 1/32 | HSAN3 mutations | Progressive sensorimotor neuropathy and sympathetic autonomic dysfunction |
| Faconi Anemia | 1/66-128 | Mutation in FANC genes | Congenital anomalies, marrow failure |
| Gaucher disease | 1/15 | Deficiency of the enzyme glucocerebrosidase | Bone pain, fractures, low platelets |
| Glycogen Storage Disease Type 1 | 1/64 | Mutations in two genes, G6PC and SLC37A4, | Severe hypoglycemia, hepatomegaly |
| Joubert Disease | 1/102 | Mutation in TMEM216 gene | Ataxia, developmental delay |
| Mucolipidosis type IV | 1/100 | Unknown - mapped to gene for mucolipin 1 | Progressive neurologic disorder |
| Maple syrup urine disease | 1/81 | Mutations in the BCKDHA, BCKDHB, and DBT genes | Early onset ketonuria, lethargy and dystonia |
| Niemann-Pick disease type A | 1/70-90 | Deficiency of acid sphingomyelinase | Hepatosplenomegaly, feeding difficulties, and loss of early motor skills – death by 2-3 |
| Tay-Sachs Disease | 1/25-30 | Deficiency in beta-Hexosaminidase A | Progressive weakness, hypotonia |
| Usher Syndrome | 1/107-165 | Mutation in MYO7A for type 1 and CLRN1 for Type 3 | Hearing loss and vision issues |

- 1. **List the carrier frequency of those conditions.**

See table above

- 1. Describe the basic mutation / genetic problem of each of those syndromes

See table above

Week 4: Pedigree analysis / patterns of inheritance

**Case 1: Please use the pedigree below to complete the case**


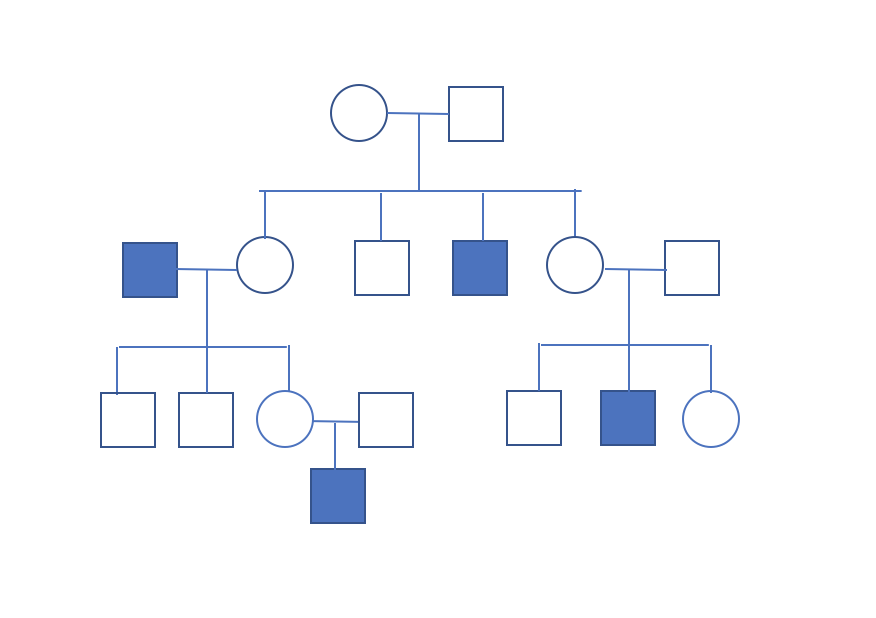


1. **A 25yo G1 comes to see you in preconception counseling. This is her pedigree.**
   1. **What type on condition are you concerned about?**

In this case, the concern is for an X-linked condition.

- - 1. **What in the pedigree makes you think this?**

In a pedigree, X-linked conditions often skip generations and affected individuals are only male.

- - 1. **What are some examples of genetic disorders that are inherited this way?**

A few examples of X-linked conditions include: Red Green color blindness, Hemophilia A and B, and Duchenne Muscular Dystrophy.

- 1. **How you counsel her about her risk of having the disorder? Could she be a carrier?**

Because this is likely an X-linked disorder, it is very unlikely that she is affected. However, there is a 50% chance that she is a carrier.

- 1. **What about a fetus? Does the gender of the fetus impact the risk?**

In X-linked conditions, fetal sex is quite important. If the patient is not a carrier, then the fetus will not be a carrier. If the patient is a carrier and her fetus is female, there is a 50% chance her fetus will be a carrier. If the fetus is male, then there is a 50% chance her fetus will be affected.

- 1. **Are there options for genetic diagnosis of a pregnancy? What are they?**

The diagnostic options depend on the condition. If there is genetic testing available, we recommend starting by performing testing on the patient for the condition. If she is not a carrier, then the evaluation ends there.

If the patient is a known carrier or tests positive for the disorder, then the diagnostic testing options vary by gestational age as discussed in week 2. However, diagnostic testing is necessary to determine fetal status. If the patient did not want diagnostic testing or was unsure due to the risks, she could consider early cfDNA to assess fetal gender. If cfDNA revealed a female fetus, then in utero genetic testing might be less useful. In contrast, if cfDNA revealed a male fetus, then a family might elect for diagnostic testing.

Case 2: Please use the pedigree below to complete the case


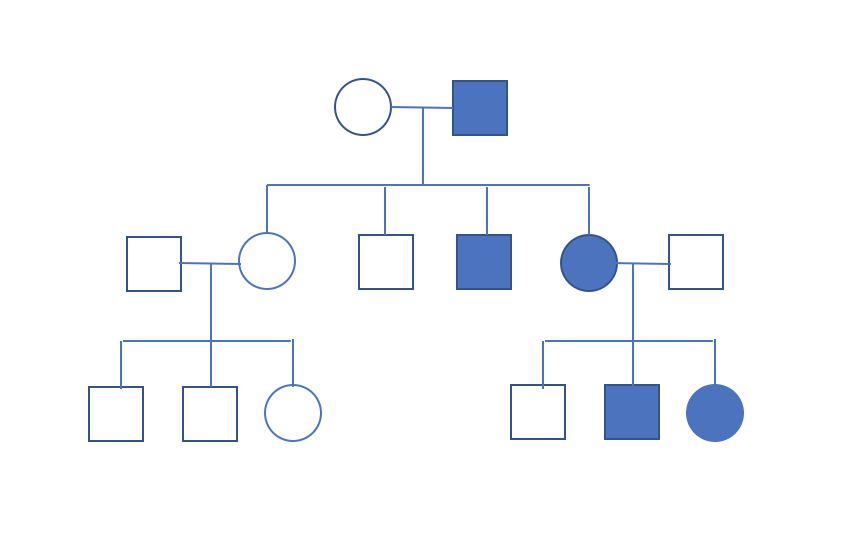


1. **A 25yo G1 comes to see you in preconception counseling. This is her partner’s pedigree (single arrow).**
   1. **What type on condition are you concerned about?**

This pedigree is consistent with an autosomal dominant (AD) condition.

- - 1. **What in the pedigree makes you think this?**

In a pedigree, an AD condition affects all generations and affects males and females equally.

- - 1. **What are some examples of genetic disorders that are inherited this way?**

A few examples of AD conditions are: Marfans syndrome, Tuberous sclerosis, and neurofibromatosis type 1.

*It is important to know that many AD disorders do not exhibit complete penetrance and may be associated with a wide variety of genetic heterogeneity. Additionally, it is some AD disorders do not present until adulthood. A common example of this is Huntington’s Disease. An in-depth discussion of these concepts (penetrance and genetic heterogeneity) is beyond the scope of this question and curriculum. However, the existence of these complex aspects of many AD diseases should be acknowledged during the conversation. Expertise of a genetic counselor should be sought in cases where patients present with pedigrees above.

- 1. **How you counsel her about partner’s risk of having the disorder? Could he be a carrier? What about a fetus?**

Based on this pedigree, because neither of her partner’s parents are affected, her partner does not have the condition. Thus, her fetus is not at risk. In AD conditions, all carriers are affected. Said another way, because her partner’s parents are not affected, he is not a carrier.

- 1. **Are there options for genetic diagnosis of a pregnancy? What are they?**

If there is a known genetic mutation, then often this can be identified using an amniocentesis. In some cases, a CVS can also be used. If the genetic mutation is not known, then diagnostic testing is more challenging, and may not be possible. For some AD conditions, mutation panels can be sent. The the case of an affected family member, testing them for the exact mutation is helpful. In those cases, the fetus can be evaluated for that specific mutation.

1. **What if her partner is the individual with the double arrow?**
   1. **How you counsel her about her fetus’ risk of having the disorder?**

In this case, there is a 50% chance that the fetus could be affected.

- 1. **Does the gender of the fetus matter?**

No, AD disorders affect males and females equally.

- 1. **Are there options for genetic diagnosis of a pregnancy? What are they?**

Depending on the condition, genetic testing options will vary. However, if there is a known mutation, then the patient could undergo either CVS or amnio depending on gestational age for diagnosis. If her partner is affected but has not been tested, he can be tested. The fetal DNA can then be assessed for his mutation.

Week 5: Cancer Genetics

Case 1: 35 yo female presents as a new patient for her GYN annual exam.

1. **You ask about her family history, which is significant for breast cancer in her mom and maternal aunts. Her maternal great aunts both also died of breast cancer.**
   1. **What are the red flags in her history regarding breast cancer risk?**

The concerning aspects of her history include a first degree relative with breast cancer and multiple family members with same kind of cancer from same side of the family.

- 1. **What other data about these cancers might be helpful?**

Other helpful information regarding breast cancer includes the age of diagnosis (pre vs post-menopausal) as well as the type of cancer (triple negative vs estrogen-receptor and progesterone-receptor positive).

- 1. **What genetic mutations are you worried about?**

BRCA 1, BRCA2 are the big ones, there are many others less common

- - 1. **How would you screen / test for these?**

There is genetic testing available for BRCA. Additionally, there are multigene panels as well. However, this patient should be referred to a Cancer genetic counselor who can specifically address this issue.

1. **After seeing you, the above patient talks to her mom more about the cancer. It turns out that her mom was tested and her mom has a BRCA2 mutation. The patient elects to be tested herself. Her test returns positive for the BRCA2 mutation.**
   1. **When does the patient need to start mammograms? What kind does she need?**

For women aged 25–29 years with known *BRCA* mutations, recommended breast cancer surveillance includes clinical breast examination every 6–12 months and annual radiographic screening (preferably, magnetic resonance imaging [MRI] with contrast). For women aged 30 years and older with known BRCA mutations or other actionable breast cancer mutations, the recommendations for breast cancer surveillance include annual mammography and annual breast MRI with contrast, often alternating every 6 months.

- 1. **Does the patient need other surveillance?**

Available screening procedures (measurement of serum CA 125 level and transvaginal ultrasonography) have not been proved to decrease the mortality rate or increase the survival rate associated with ovarian cancer in high-risk populations, such as those with BRCA mutations.

- 1. **How would you counsel the patient about her risks of breast cancer? Other cancers?**

*BRCA1 or 2* carriers have a 45-85 percent chance of developing breast cancer by age 70. The risk of ovarian cancer is 35-70% for BRCA1 and 10-30% for BRCA2. Given the complexity of these diagnoses, patients should be referred to a cancer genetic counselor for help in navigating testing and the medical ramifications of these diagnoses.

- 1. **How would you counsel the patient regarding management of her ovaries / tubes?**

ACOG recommends a risk-reducing bilateral salpingo-oophorectomy in women with BRCA2 mutations after fertility is complete. This reduces risk of ovarian cancer by about 80%. However, given the complexities of this decision, we recommend referral to genetic counseling and shared decision making.

1. **If her mom was tested and was negative for a BRCA mutation, what breast cancer screening regimen would you recommend for your patient?**

She is managed based on family history.

Case 2: 53yo comes in her for her annual exam

1. **In the last year, she had a maternal aunt diagnosed with endometrial cancer and her mother died of colon cancer.**
   1. **What other family history might be helpful in figuring out if there is a hereditary cancel syndrome present?**

We would be curious if any other family members had been diagnosed with cancer or had died of cancer.

- - 1. **What cancer syndromes are on your differential diagnosis?**

In this case, the most concerning is Lynch syndrome (aka hereditary nonpolyposis colorectal cancer (HNPCC).

- - 1. **Describe each of these, what cancers are seen, and what are the molecular mechanisms of these syndromes? How would you diagnose each of these?**

Lynch syndrome can present with colon cancer as well as endometrial cancer. It is due to mutations in the mismatch repair genes. The patient herself or her tumor can be tested.

- - 1. **What screening would you recommend for her if she does not appear to have a hereditary cancer syndrome?**

Routine colon cancer screening

- - 1. If she does have Lynch syndrome?

For individuals with Lynch syndrome, screening imlcudes colonoscopy every 1–2 years, beginning at age 20–25 years, or 2–5 years before the earliest cancer diagnosis in the family, whichever is earlier. It also includes endometrial biopsy every 1–2 years, beginning at age 30–35 years.
