# Supplementary material for: Genetics for the Women's Health Trainee: A Five-Module Curriculum
Source: MedEdPORTAL. 2019 Jan 18;15:10797. doi: 10.15766/mep_2374-8265.10797 (PMC6376891; doi:10.15766/mep_2374-8265.10797)
Supplement: Supplementary file 1 — A. Welcome Email.docx B. Objectives and Readings.docx C. Cases Only.docx D. Cases With Answers.docx E. CREOG Objectives.docx F. ACGME Milestones.docx G. End-of-Modules Feedback Form.docx [file mep-15-10797-s001.zip › E. CREOG Objectives.docx]

Appendix E: CREOG objectives^1^

**Objectives listed by module**

**Module 1: Prenatal Screening for Aneuploidy**

*Unit 1: General Considerations*

I: Basic Science

B. Describe patterns of inheritance

2. Non-mendelian Modes

IV: Non-clinical overlapping content

A: Communication

3. Describe the difference between a screening test and a diagnostic test

*Unit 5: Specialty Objectives*

II: Maternal-Fetal Medicine

H: Genetics

1. Solicit a family pedigree
2. Describe and perform preconception counseling and testing
3. Describe and perform antenatal testing and counseling

*Unit 6: Procedures in OBGYN*

- Amniocentesis – genetic diagnosis: Understand
- Chorionic villus sampling: Understand

**Module 2: Prenatal Diagnostic Testing**

Unit 1: General Considerations

IV: Non-clinical overlapping content

A: Communication

3. Describe the difference between a screening test and a diagnostic test

Unit 5: Specialty Objectives

II: Maternal-Fetal Medicine

H: Genetics

1. Solicit a family pedigree
2. Describe and perform preconception counseling and testing
3. Describe and perform antenatal testing and counseling

*Unit 6: Procedures in OBGYN*

- Amniocentesis – genetic diagnosis: Understand
- Chorionic villus sampling: Understand

**Module 3: Prenatal Carrier Screening**

*Unit 1: General Considerations*

I: Basic Science

B. Describe patterns of inheritance

1. Mendelian Modes

2. Non-mendelian Modes

IV: Non-clinical overlapping content

A: Communication

3. Describe the difference between a screening test and a diagnostic test

*Unit 5: Specialty Objectives*

II: Maternal-Fetal Medicine

H: Genetics

1. Solicit a family pedigree
2. Describe and perform preconception counseling and testing
3. Describe and perform antenatal testing and counseling

*Unit 6: Procedures in OBGYN*

- Amniocentesis – genetic diagnosis: Understand
- Chorionic villus sampling: Understand

**Module 4: Pedigrees**

*Unit 1: General Considerations*

I: Basic Science

B. Describe patterns of inheritance

1. Mendelian Modes

2. Non-mendelian Modes

*Unit 5: Specialty Objectives*

II: Maternal-Fetal Medicine

H: Genetics

1. Solicit a family pedigree
2. Describe and perform preconception counseling and testing
3. Describe and perform antenatal testing and counseling
   1. Preform prenatal screening
   2. Refer for Diagnostic testing

**Module 5: Cancer Genetics**

*Unit 5: Specialty Objectives*

II: Maternal-Fetal Medicine

H: Genetics

1. Solicit a family pedigree
2. Describe and perform preconception counseling and testing

III: Oncology

A: Evaluate and mitigate the risks of genetic predisposition to cancer

1. Discuss results of genetic screening for cancer in patients with a family history
   1. Genetic Markers (*BRCA1* and *BRCA2*)
   2. Negative testing
   3. Indications for referral to a genetic counselor

**Optional Activities:**

**Sit in on sessions with a Prenatal Genetic Counselor**

*Unit 1: General Considerations*

IV: Non-clinical overlapping content

A: Communication

3. Describe the difference between a screening test and a diagnostic test

C: Work with health care professionals in multidisciplinary teams

2. Communicate effectively with others as a member or leader of a health care team

4. Develop awareness of the roles and effects of other health care professionals, the health care organization and society on patient care

*Unit 5: Specialty Objectives*

II: Maternal-Fetal Medicine

H: Genetics

1. Solicit a family pedigree
2. Describe and perform preconception counseling and testing
3. Describe and perform antenatal testing and counseling
   1. Preform prenatal screening
   2. Refer for Diagnostic testing

**Sit in on sessions with a Cancer Genetic Counselor**

*Unit 1: General Considerations*

IV: Non-clinical overlapping content

A: Communication

3. Describe the difference between a screening test and a diagnostic test

C: Work with health care professionals in multidisciplinary teams

2. Communicate effectively with others as a member or leader of a health care team

4. Develop awareness of the roles and effects of other health care professionals, the health care organization and society on patient care

*Unit 5: Specialty Objectives*

II: Maternal-Fetal Medicine

H: Genetics

1. Solicit a family pedigree
2. Describe and perform preconception counseling and testing

III: Oncology

A: Evaluate and mitigate the risks of genetic predisposition to cancer

1. Discuss results of genetic screening for cancer in patients with a family history
   1. Genetic Markers (*BRCA1* and *BRCA2*)
   2. Negative testing
   3. Indications for referral to a genetic counselor

References

1. *CREOG Educational Objectives: A Core Curriculum in Obstetrics and Gynecology.* 10th Edition ed. Washington DC: American College of Obstetricians and Gynecologists; 2013.
