# Supplementary material for: Genetics for the Women's Health Trainee: A Five-Module Curriculum
Source: MedEdPORTAL. 2019 Jan 18;15:10797. doi: 10.15766/mep_2374-8265.10797 (PMC6376891; doi:10.15766/mep_2374-8265.10797)
Supplement: Supplementary file 1 — A. Welcome Email.docx B. Objectives and Readings.docx C. Cases Only.docx D. Cases With Answers.docx E. CREOG Objectives.docx F. ACGME Milestones.docx G. End-of-Modules Feedback Form.docx [file mep-15-10797-s001.zip › F. ACGME Milestones.docx]

**Appendix F: ACGME Milestones**

**Below is a list of the milestones that we have been able to evaluate at our institution, as the modules are used during a 6 week rotation. Because our learners are interns, most of them somewhere around Level 2 (which are the specifics listed here).

We have annotated those milestones which are better assessed if there are opportunities for observing / working with genetic counselors during the use of the modules with (β).

Antepartum Care and Complications of Pregnancy — Patient Care

- Provides complete antepartum care for women with uncomplicated pregnancies (Level 2)
  - **Important – This milestone must be interpreted in context – as we are considering counseling for genetic issues in pregnancy as PART of the antepartum care. Thus, if you assess this milestone, it must be in context of other prenatal care settings

Compassion, Integrity, and Respect for Others — Professionalism

- Consistently shows compassion, integrity, and respect in typical situations with patients, peers, and members of the health care team (Level 2)
- Consistently demonstrates sensitivity and responsiveness to diversity of patients’ ages, cultures, races, religions, abilities, or sexual orientations (Level 2) (β)
- Accepts constructive feedback to improve his or her ability to demonstrate compassion, integrity, and respect for others (Level 2)

Accountability and Responsiveness to the Needs of Patients, Society, and the Profession — Professionalism

- Is consistently punctual for clinical assignments and responsive to requests for assistance; completes administrative duties (e.g., medical records, reports) on time and without reminders (Level 2)

Respect for Patient Privacy, Autonomy, Patient-Physician Relationship — Professionalism

- Demonstrates an understanding of ethical principles, including boundary issues, and consciously applies them in patient care (Level 2)

Communication with Physicians and Other Health Professionals and Teamwork — Interpersonal and Communication Skills

- Demonstrates an understanding of the roles of health care team members, and communicates effectively within the team (Level 2) (β)

Informed Consent and Shared Decision Making — Interpersonal and Communication Skills

- Begins to engage patients in shared decision making, and obtains informed consent for basic procedures (Level 2)
  - **This is better assessed if the learner has a chance to observe diagnostic testing as well as genetic counseling sessions
