# Supplementary material for: Genetics for the Women's Health Trainee: A Five-Module Curriculum
Source: MedEdPORTAL. 2019 Jan 18;15:10797. doi: 10.15766/mep_2374-8265.10797 (PMC6376891; doi:10.15766/mep_2374-8265.10797)
Supplement: Supplementary file 1 — A. Welcome Email.docx B. Objectives and Readings.docx C. Cases Only.docx D. Cases With Answers.docx E. CREOG Objectives.docx F. ACGME Milestones.docx G. End-of-Modules Feedback Form.docx [file mep-15-10797-s001.zip › G. End-of-Modules Feedback Form.docx]

Genetics feedback

Start of Block: Default Question Block

Q1 Please select the response that best describes your thoughts on the genetics curriculum

|  | Strongly agree (1) | Somewhat agree (2) | Neither agree nor disagree (3) | Somewhat disagree (4) | Strongly disagree (5) |
| --- | --- | --- | --- | --- | --- |
| The genetics curriculum was helpful in increasing my knowledge of prenatal genetics (1) |  |  |  |  |  |
| The genetics curriculum was helpful in increasing my knowledge of CANCER genetics (2) |  |  |  |  |  |
| I would recommend curriculum this to other residents (3) |  |  |  |  |  |
| After completing the genetics curriculum, I felt more comfortable counseling patients about screening options for aneuploidy (4) |  |  |  |  |  |
| After completing the genetics curriculum, I felt more comfortable counseling patients about diagnostic testing for aneuploidy (5) |  |  |  |  |  |
| This curriculum helped me do better on the CREOG exam (6) |  |  |  |  |  |
| The discussion format was a good way to learn (7) |  |  |  |  |  |
| The articles were helpful (8) |  |  |  |  |  |

Q2 Please list one thing that would improve the genetics curriculum

________________________________________________________________

End of Block: Default Question Block
